# Comparing Federal Communications Commission and Microsoft Estimates of Broadband Access for Mental Health Video Telemedicine Among Veterans: Retrospective Cohort Study

**DOI:** 10.2196/47100

**Published:** 2024-08-08

**Authors:** Amy MJ O'Shea, Kailey Mulligan, Knute D Carter, Bjarni Haraldsson, Charlie M Wray, Ariana Shahnazi, Peter J Kaboli

**Affiliations:** 1 Center for Access and Delivery Research and Evaluation (CADRE) Iowa City VA Healthcare System Iowa City, IA United States; 2 Veterans Rural Health Resource Center-Iowa City VA Office of Rural Health Iowa City, IA United States; 3 Department of Internal Medicine University of Iowa Carver College of Medicine Iowa City, IA United States; 4 Department of Biostatistics University of Iowa College of Public Health Iowa City, IA United States; 5 Department of Medicine University of California San Francisco, CA United States; 6 Section of Hospital Medicine San Francisco Veterans Affairs Medical Center San Francisco, CA United States

**Keywords:** broadband, telemedicine, Federal Communications Commission, veterans, United States Department of Veterans Affairs, internet, mental health care, veteran health, broadband access, web-based, digital

## Abstract

**Background:**

The COVID-19 pandemic highlighted the importance of telemedicine in health care. However, video telemedicine requires adequate broadband internet speeds. As video-based telemedicine grows, variations in broadband access must be accurately measured and characterized.

**Objective:**

This study aims to compare the Federal Communications Commission (FCC) and Microsoft US broadband use data sources to measure county-level broadband access among veterans receiving mental health care from the Veterans Health Administration (VHA).

**Methods:**

Retrospective observational cohort study using administrative data to identify mental health visits from January 1, 2019, to December 31, 2020, among 1161 VHA mental health clinics. The exposure is county-level broadband percentages calculated as the percentage of the county population with access to adequate broadband speeds (ie, download >25 megabits per second) as measured by the FCC and Microsoft. All veterans receiving VHA mental health services during the study period were included and categorized based on their use of video mental health visits. Broadband access was compared between and within data sources, stratified by video versus no video telemedicine use.

**Results:**

Over the 2-year study period, 1,474,024 veterans with VHA mental health visits were identified. Average broadband percentages varied by source (FCC mean 91.3%, SD 12.5% vs Microsoft mean 48.2%, SD 18.1%; *P*<.001). Within each data source, broadband percentages generally increased from 2019 to 2020. Adjusted regression analyses estimated the change after pandemic onset versus before the pandemic in quarterly county-based mental health visit counts at prespecified broadband percentages. Using FCC model estimates, given all other covariates are constant and assuming an FCC percentage set at 70%, the incidence rate ratio (IRR) of county-level quarterly mental video visits during the COVID-19 pandemic was 6.81 times (95% CI 6.49-7.13) the rate before the pandemic. In comparison, the model using Microsoft data exhibited a stronger association (IRR 7.28; 95% CI 6.78-7.81). This relationship held across all broadband access levels assessed.

**Conclusions:**

This study found FCC broadband data estimated higher and less variable county-level broadband percentages compared to those estimated using Microsoft data. Regardless of the data source, veterans without mental health video visits lived in counties with lower broadband access, highlighting the need for accurate broadband speeds to prioritize infrastructure and intervention development based on the greatest community-level impacts. Future work should link broadband access to differences in clinical outcomes.

## Introduction

During the COVID-19 pandemic, in-person visits were heavily restricted, allowing for the rapid expansion of video-based telemedicine. With substantial prepandemic investments in training and technology, the Veterans Health Administration (VHA) was well positioned to quickly expand the use of video visits during the pandemic [1]. Within VHA, video-based visits are delivered primarily through Veterans Affairs (VA) Video Connect, a secure telemedicine app that connects VHA providers and patients via live video at a location of the veteran’s choosing (eg, home and work).

However, video telemedicine requires adequate broadband speeds, defined by the Federal Communications Commission (FCC) and for this study as ≥25 megabits per second (Mbps) download and ≥3 Mbps upload speeds [2]. As broadband services expand, some have argued higher broadband speeds are essential for daily life, including health care services [3,4]. Beyond infrastructural availability, access to adequate broadband internet is influenced by socioeconomic factors, age, digital literacy, and rurality [5-9]. Homes in rural areas are less likely to have access to terrestrial broadband infrastructure [9,10]. This is amplified among veterans, of whom 30% reside in rural areas and 15% lack home internet [11,12]. Previous research has shown that rural, homeless, and older populations typically experience fewer telehealth visits [13,14]. Altogether, these individual- and area-level characteristics and their relationship with broadband access may exacerbate disparities for already underserved or medically vulnerable populations [15,16].

One of the most challenging and critical tasks in telemedicine research is to define broadband access. In the United States, the most common publicly available data comes from the FCC and is reported directly by fixed broadband internet providers on a biannual basis [17-21]. These data include the number of providers, speeds, and technologies offered within a census block. Although this detail can make the data prohibitively large, they are longitudinally available nationally and by state. Additionally, in the timeframe of this research, it is well known that the FCC data likely overrepresent broadband coverage as internet service providers report speed and technology combinations aggregated at the census block [22]. Alternatively, the Connect2Health platform links December 2015 broadband data with health and demographic data from a variety of sources in a preprocessed, ready-to-use structure [10,23], although these data have become somewhat dated.

Beyond the FCC, the publicly available Microsoft US broadband use data measure the percentage of a county’s population using the internet at a minimum download speed of 25 Mbps [19]. The FCC data originate from internet providers (eg, Comcast, Mediacom) and can be considered a measure of availability, whereas the Microsoft data aggregate broadband speeds people are actively using, illustrating broadband accessibility. This can be thought of as a difference between an ideal internet landscape without individual-level influences (FCC) versus actual use (Microsoft), which is inherently influenced by unmeasurable outside factors. This is especially true as reports to the FCC during our study period required broadband providers to report census blocks where a given technology and speed was delivered to at least 1 location, not necessarily the entire census block. This means that FCC-defined broadband availability may not be universally or equally available to all residents of a census block [9]. Although Microsoft data improve upon the measurement of “real” access, there are also several limitations. Most importantly, these data require Microsoft products to be in use, which may bias results toward those able to afford a Microsoft license. In the same regard, this “downstream” measurement may not reflect the highest speed one could purchase due to perceived speed needs, desirability, or affordability issues. Finally, other issues such as internet traffic, user download bandwidth limits, and technology type (eg, digital subscriber line, cable modem, and fiber) may lead to discrepancies in broadband speed measurement.

To our knowledge, only 1 published research study has compared the divergence between the FCC and Microsoft broadband measures to assess the relationship between county-level social vulnerability and broadband access [24]. The goal of this study adds to this effort by comparing (1) FCC and (2) Microsoft estimates of broadband access and further by applying both broadband measures to the evaluation of VHA mental health video visit use as a case study of how the definition of broadband access may impact health services research. We hypothesized that (1) higher broadband access would be estimated using FCC versus Microsoft data and (2) veterans living in counties with lower broadband access would have fewer video mental health visits. Identifying how the definition of broadband speeds may influence research conclusions will inform the development of more robust research methodologies, aid in developing targeted health system initiatives to reduce the digital divide, and ultimately improve patient access to care. Accurate measurement at sufficiently granular and temporal levels is critical for telemedicine research, as it has implications for broadband improvements, government policy, health care, work, and day-to-day life in the digital age.

## Methods

### Study Design and Data Sources

This is a retrospective, cross-sectional, observational, cohort study of VHA mental health outpatient visits between January 1, 2019, and December 31, 2020. We chose mental health care as a case study of how 2 different national broadband measures may impact the assessment of the association between broadband access and telemedicine use because studies have shown that 58% of outpatient mental health visits in VHA are provided through video-based means [15]. This study followed the STROBE (Strengthening the Reporting of Observational Studies in Epidemiology) reporting guideline [25].

Patient-level data, including patient demographics and outpatient mental health visits, were obtained from the Corporate Data Warehouse within the VA Informatics and Computing Infrastructure, an integrated electronic health records system including VHA electronic health records and administrative data. The 2010 Census Bureau TIGER/Line shapefile contains geographic entity codes (ie, census block, census block group, and county) [26]. Broadband access was obtained at the census block, the smallest geography of the US Census Bureau, for FCC fixed broadband data and at the county level for Microsoft US broadband use data [27]. The area deprivation index (ADI), a measure of neighborhood socioeconomic disadvantage, is reported in the census block group [28]. Both broadband and ADI data were spatially merged with the census shapefiles to overlay the latitude and longitude of each veteran’s home address within a year to either the census block polygon for FCC broadband, the county polygon for Microsoft use data, or the census block group for the ADI. Population estimates are from the 2019 and 2020 census block-level estimates created by FCC staff based on public data from the US Census Bureau [29], while the estimate of veteran enrollees residing in each county is from the VA National Center for Veterans Analysis and Statistics [30].

### Patient Population

The study cohort includes veterans aged 18 and older with a US home address having at least 1 VHA mental health visit between January 1, 2019, and December 31, 2020. Visits were classified as occurring in person, by telephone, or by video. All veterans receiving VHA mental health services during the study period were categorized based on their history of ever using video-based mental health care during the study period. The video cohort is thus all veterans who experienced 1 or more video-based mental health visits. We excluded care received at residential rehabilitation centers, nursing homes, or domiciliary visits, as well as care received in US territories and freely associated states.

### Broadband Measures

Our primary exposure was nationwide county-level broadband access identified by (1) FCC or (2) Microsoft. FCC fixed broadband data originate from internet service providers, who report available broadband speeds by census block and technology type to the FCC. These data are available biannually in June and December. A total of 2 reporting timeframes were selected: December 2019 [31] and June 2020 [32]. To match the format of the Microsoft data, FCC data were aggregated at the county level (Multimedia Appendix 1), excluding satellite technology. Satellite technology was excluded because although it is widely available at the time of this study, it suffers from interference and delays, has relatively low subscription rates, and could be prohibitively expensive [22,33,34]. To be considered adequate, FCC data were required to demonstrate ≥25 Mbps download and ≥3 Mbps upload speeds, in line with speed recommendations for videoconferencing [2]. This FCC availability percentage can be interpreted as the percent of the county population (ie, 0%-100%) with access to adequate fixed terrestrial broadband download and upload speeds.

The Microsoft data originate from the Microsoft Airband Initiative and report the percent of each county’s population with download speeds >25 Mbps as measured using the time required to download Microsoft products and updates [27]. Due to the nature of software updates, upload speeds could not be assessed. We note this is slightly different than the calculation using FCC data, which uses both download and upload speeds to determine speed adequacy. The Microsoft data are available at 2 time points: November 2019 and October 2020. This Microsoft use percentage represents the percent of the county population (ie, 0%-100%) using adequate download broadband speeds (ie, >25 Mbps).

For both FCC and Microsoft data, broadband access percentages were matched to patient-level data by county using the latitude and longitude of the veteran’s residential address, matched to the timeframe of the broadband data (eg, 2019 or 2020).

### Video Visit Outcome

Mental health clinic visits were assessed by clinic stop codes with visits categorized as occurring either in-person, by telephone, or via video-based care. Each patient’s mental health visits per quarter were identified, by modality and overall, and then aggregated by county of residence. Visits were also categorized as occurring prepandemic (from January 2019 to March 2020) or postpandemic onset (from April 2020 to December 2020).

### Covariates

Patient demographics included age (as of March 2020), sex, race, ethnicity, and patient rurality. Patient rurality was identified using the geocoded location of the patient’s home and dichotomized into urban and rural (ie, rural and highly rural) according to rural-urban commuting area classifications [35]. Race was categorized as Black or African American, other (including American Indian or Alaska Native, Asian, Multiracial, or Pacific Islander), and White. Ethnicity was reported as being Hispanic or not Hispanic. The patient census block was updated quarterly, based on the veteran residential location. Patient demographics and rurality were also summarized at the county level as a percentage.

### Statistical Analysis

Initially, we describe the cohort of patients receiving VHA mental health care stratified by their use of mental health video visits during the study period (ie, video and no video cohorts). To better understand the differences between FCC-based broadband availability and Microsoft-based broadband use, we compare differences over time within and between data sources using empirical cumulative distribution functions. These functions plot the broadband percentage on the x*-*axis and the cumulative percent of veterans living in a county with the corresponding broadband percentage on the y-axis. Similar plots illustrate FCC and Microsoft broadband percentages stratified by veteran rurality (ie, rural or urban) and video cohort status (ie, at least 1 video visit in the study period or not).

Mental health visit rates in 2019 and 2020 were visualized within the COVID-19 pandemic period by video cohort status and broadband access categorized into quartiles (ie, cutoffs at 25%, 50%, and 75%). Finally, to better understand the association between quarterly mental health visit rates and broadband access leading into and during the COVID-19 pandemic, we quantify this association using a multivariate generalized estimating equation with a negative binomial distribution, log link function, and an offset for the county population. A total of 2 models are generated: 1 using each broadband measure. Broadband access was aligned by study year (eg, 2019 or 2020). These models were adjusted for patient characteristics as listed previously; included a binary indicator variable for time before and after the onset of the COVID-19 pandemic, broadband percentage, and the interaction of these 2 variables; and implemented random effects to account for repeated measurement within the county.

The authors had full access to and take full responsibility for the integrity of the data. All analyses were conducted using the SAS Enterprise Guide (version 8.2, 64-bit; SAS Institute).

### Ethical Considerations

This work was completed as a quality improvement evaluation and was designated as nonresearch by the VHA Office of Connected Care (COR 20-199-05), the University of Iowa institutional review board (#202010441), and the VA Research Administration. This VHA documentation ensures work was not carried out under a human participants protocol and is part of institutionally sanctioned quality improvement activities. This study was conducted without direct patient contact using data routinely collected in the electronic health record and of minimal risk; therefore, a waiver of informed consent was obtained.

## Results

### Patient Population

Over the 2-year study period, 1,474,024 veterans were seen across 1161 VHA mental health clinics. Of these, 418,898 (28.4%) participated in at least 1 video visit in the study period (ie, video cohort). The video cohort compared to those without a video visit was less likely to be rural (115,421/418,898, 27.6% vs 346,945/1,055,126, 32.9%; *P*<.001) and more likely to be female (90,677/418,898, 21.7% vs 130,350/1,055,126, 12.4%; *P*<.001; Table 1). Further, veterans in the video cohort, on average, displayed higher broadband availability via FCC data (92.3% vs 91%; *P*<.001) and higher broadband use via Microsoft data (50.8% vs 47.1%; *P*<.001) in 2019. Similar differences were detected in 2020.

**Table 1:** Veteran demographics and broadband percentage by data source overall and by mental health video visit status.

Category				Overall cohort (n=1,474,024)		Video cohort^a^ (n=418,898)		No video cohort (n=1,055,126)
Ever rural residence^b^, n (%)				462,366 (31.4)		115,421 (27.6)		346,945 (32.9)
Age (years), mean (SD)				55.4 (16.0)		50.4 (14.9)		57.4 (16.0)
**Sex, n(%)**								
	Female			221,027 (15)		90,677 (21.6)		130,350 (12.4)
	Male			1,252,602 (85)		328,158 (78.4)		924,454 (87.6)
**Race^c^, n(%)**								
	Black or African American			329,879 (22.4)		94,616 (22.6)		235,263 (22.3)
	White			989,465 (67.1)		274,971 (65.6)		714,494 (67.7)
	Other			65,239 (4.4)		21,728 (5.2)		43,511 (4.1)
	Unknown or missing			89,441 (6.1)		27,583 (6.6)		61,858 (5.9)
**Ethnicity^d^, n (%)**								
	Hispanic or Latino			115,063 (7.8)		40,897 (9.8)		74,166 (7)
	Not Hispanic or Latino			1,305,467 (88.6)		361,968 (86.4)		943,499 (89.4)
	Other			53,494 (3.6)		16,033 (3.8)		37,461 (3.6)
**ADI^e^, mean (SD)**								
	National rank			53.9 (25.3)		50.1 (24.8)		55.4 (25.4)
	State rank			5.6 (2.7)		5.3 (2.7)		5.7 (2.7)
**FCC^f^ broadband availability (%)**								
	**Mean (SD)**							
		2019	91.3 (12.5)		92.3 (11.2)		91.0 (12.9)	
		2020	92.3 (11.2)		93.1 (10.1)		92.0 (11.6)	
	**Median (IQR)**							
		2019	95.7 (8.1)		96.0 (6.5)		95.7 (8.8)	
		2020	96.2 (6.9)		96.4 (6.1)		96.2 (7.6)	
**Microsoft broadband use (%)**								
	**Mean (SD)**							
		2019	48.2 (18.1)		50.8 (17.4)		47.1 (18.2)	
		2020	61.4 (18.6)		64.2 (17.5)		60.2 (18.9)	
	**Median (IQR)**							
		2019	51.0 (26.0)		54.0 (24.0)		49.0 (26.0)	
		2020	64.7 (23.4)		68.5 (22.4)		64.1 (23.4)	

^a^Differences between the video and no video cohorts are significant at *P*<.001 for all variables, where continuous variables were assessed using a 2-tailed *t* test and categorical variable using a two-sided chi-square test.

^b^Includes patients who lived in a rural area at any time during the study period.

^c^Other races include American Indian or Alaskan Native, Asian, Multiracial, or Pacific Islander categories.

^d^Other ethnicity includes missing, multiple ethnicity, and unknown categories.

^e^ADI: area deprivation index; ranks neighborhoods by socioeconomic disadvantage on a scale of 0-100 with lower rankings indicating less social disadvantage.

^f^FCC: Federal Communications Commission.

### Broadband Measures

**Figure 1:**
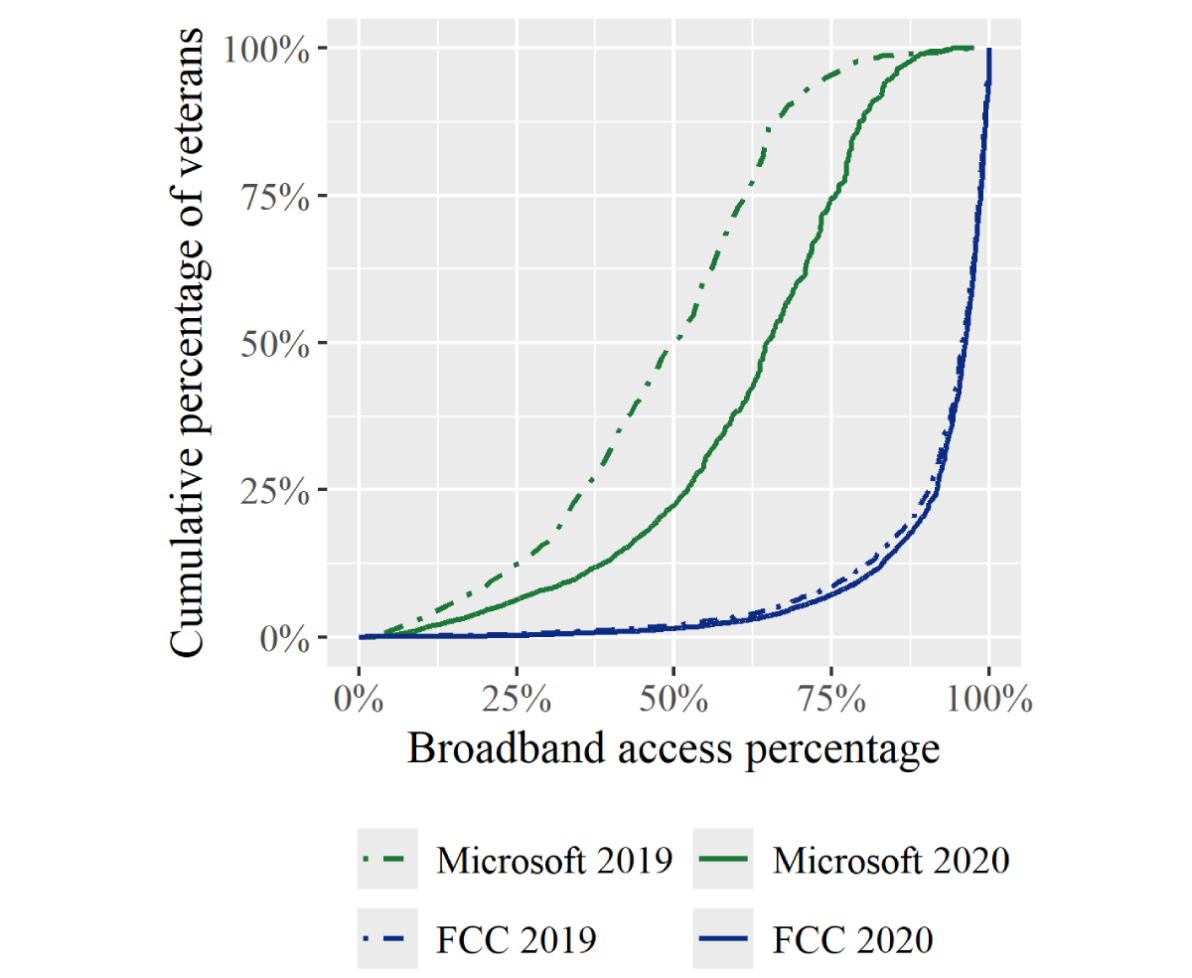
Empirical cumulative distribution functions of broadband penetration rates among veterans using mental health services by data set and year. FCC: Federal Communications Commission.

Next, we plotted the empirical cumulative distribution functions, which represent the cumulative percent of veterans living in a county with a specified broadband percentage, split by data set and year (Figure 1). For example, comparing the 2020 data from FCC (blue) and Microsoft (green), 1.4% (21,338/1,474,024) versus 22.4% (n=327,584/1,474024) of veterans live in a county with a broadband percentage of 50% or less. We observed an overall increase in both broadband availability and use, although increases in use were more pronounced.

### Video Visit Outcome

**Figure 2:**
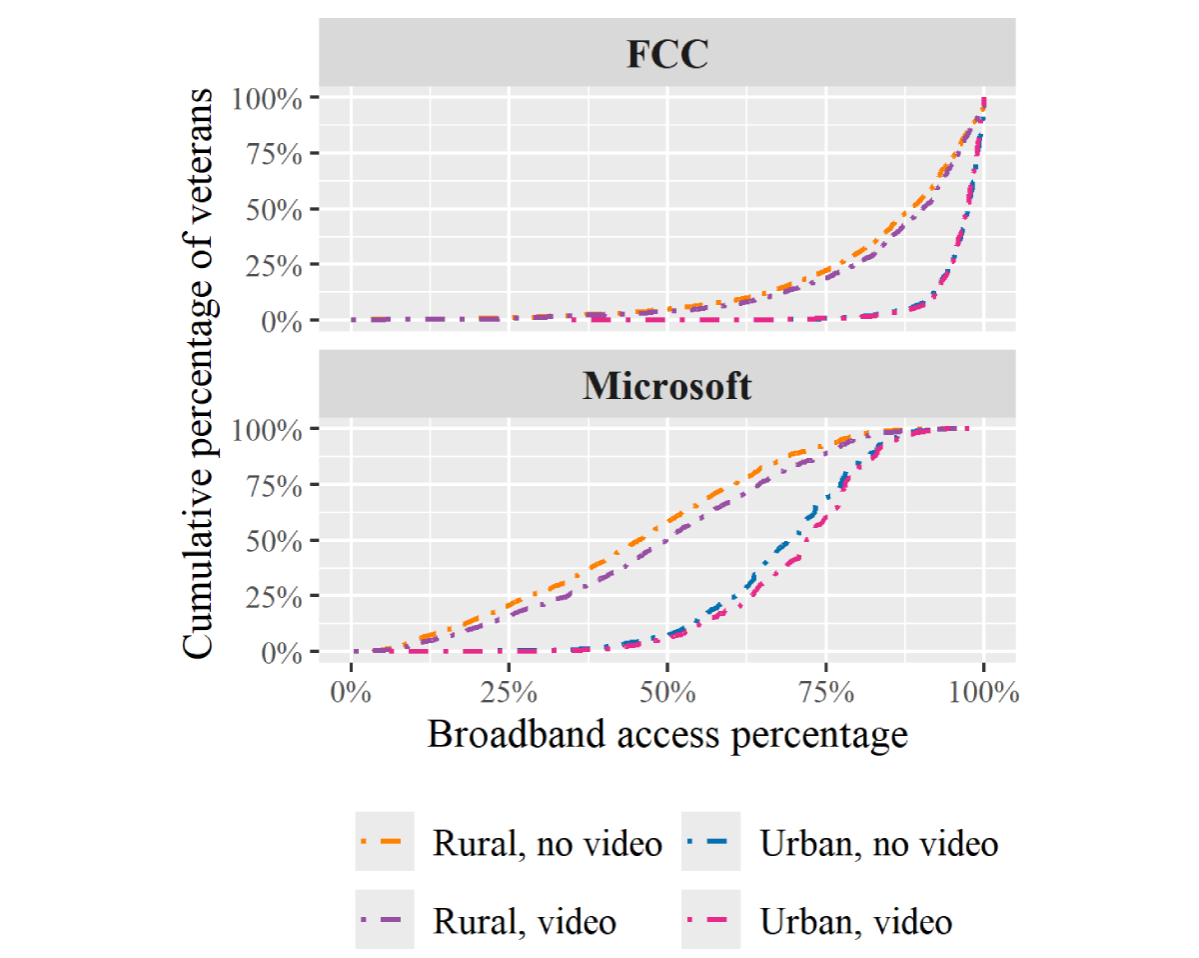
Empirical cumulative distribution functions of veteran penetration rates of 2020 data set stratified by video cohort. FCC: Federal Communications Commission.

Stratifying the 2020 veteran broadband percentages by video cohort and rurality (Figure 2) we note that, compared to veterans without video visits, veterans who had at least 1 video visit had greater broadband access. Regardless of broadband data source, veterans in urban areas had higher broadband percentages than those living in rural areas.

Similarly, as broadband access increased, a higher proportion of veterans used video visits (Multimedia Appendix 2). Based on our findings, we estimate that of the 19.2 million living veterans in 2020, between 1.4 million (7.3%) and 7.2 million (37.4%) veterans do not have access to sufficient broadband to participate in video telemedicine (Multimedia Appendix 3).

**Figure 3:**
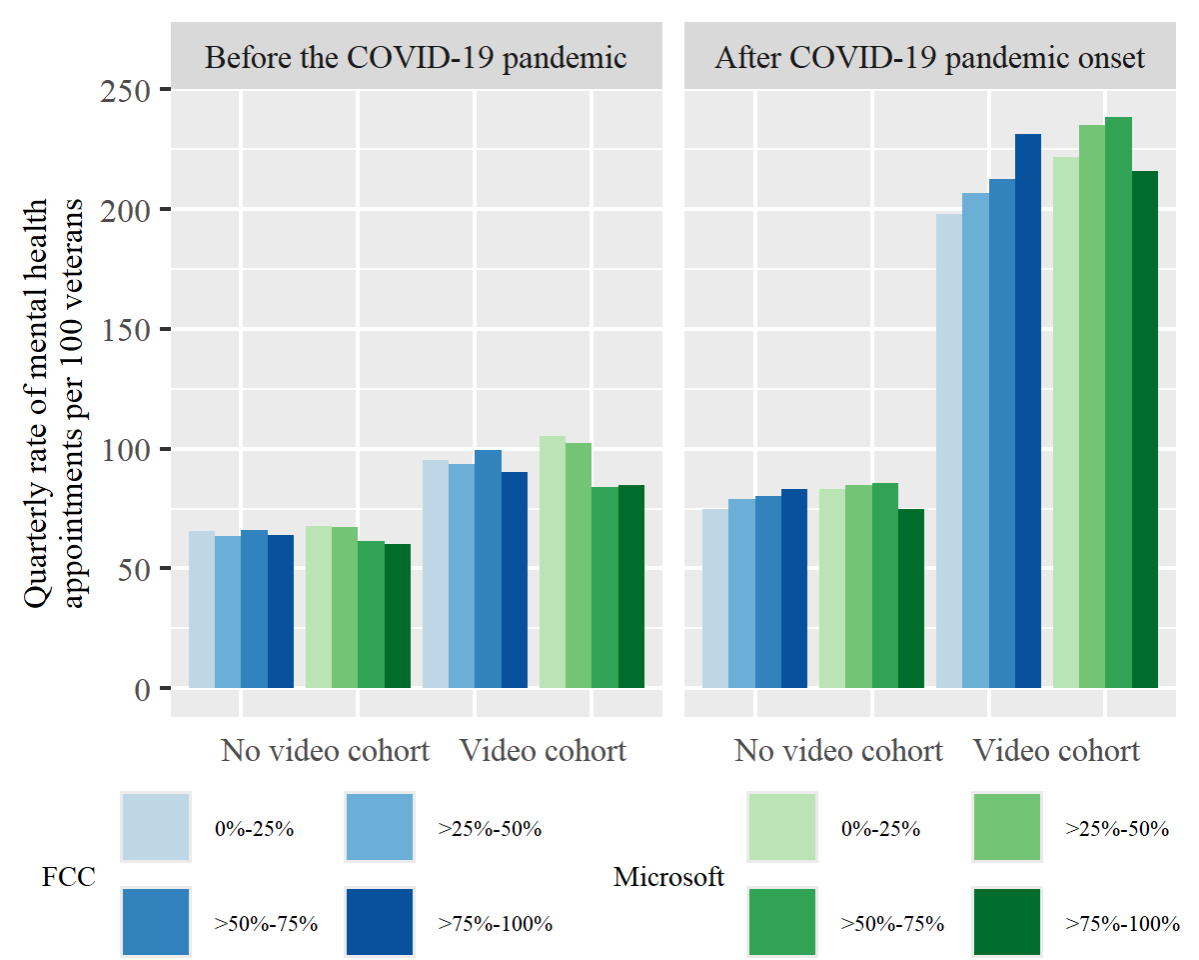
Overall quarterly mental health visit rate by data set, penetration rate, video cohort, and pandemic time period. FCC: Federal Communications Commission.

After the onset of the COVID-19 pandemic, there has been a clear association between group and mental health visit use, where those living in a county with a higher broadband percentage have a higher mental health visit rate (Figure 3). Both before and during the COVID-19 pandemic, the video cohort had a higher quarterly rate of mental health visits compared to the nonvideo cohort. Furthermore, among the video cohort, the mental health visit rate doubled compared to before the COVID-19 pandemic.

Finally, 2 longitudinal multivariate generalized estimating equation models were developed identically but used either FCC or Microsoft broadband percentages to evaluate the association between broadband access and quarterly mental health video visit use (Table 2).

**Table 2:** Multivariate negative binomial regression models using generalized estimating equations to predict quarterly mental health video visit rate by county for each broadband data source.

Pandemic indicator (after vs before)^a^			Federal Communications Commission model; IRR^b^ (95% CI)		Microsoft broadband use model; IRR (95% CI)
**At broadband percentage (%)**					
	10	5.74 (4.85-6.80)		6.05 (5.67-6.45)	
	20	5.91 (5.10-6.84)		6.24 (5.93-6.57)	
	30	6.08 (5.36-6.89)		6.43 (6.16-6.72)	
	40	6.25 (5.64-6.93)		6.64 (6.36-6.92)	
	50	6.43 (5.92-6.98)		6.84 (6.53-7.17)	
	60	6.62 (6.21-7.04)		7.06 (6.66-7.48)	
	70	6.81 (6.49-7.13)		7.28 (6.78-7.81)	
	80	7.00 (6.74-7.28)		7.51 (6.89-8.18)	
	90	7.20 (6.90-7.51)		7.74 (6.99-8.57)	

^a^The models were adjusted for rural residency, race, and Hispanic ethnicity summarized at the county level as percentages, as well as average county-level area deprivation index and age.

^b^IRR: incidence rate ratio.

Using FCC model estimates, given all other covariates are constant and assuming the FCC broadband percentage was set at 70%, the incident rate ratio (IRR) of county-level quarterly mental health video visits during the pandemic was 6.81 times (95% CI 6.49-7.13) the rate before the pandemic. In comparison, the Microsoft model estimates a higher rate of mental health video visits during versus before the pandemic at the same broadband percentage (IRR 7.28; 95% CI 6.78-7.81). This was the case for all broadband percentages assessed.

## Discussion

### Principal Findings

In this study, we found that (1) the measurement of county-level broadband access varied by broadband data source; (2) regardless of data source, overall, adequate broadband access improved over time, although these gains were most pronounced in the measurement of Microsoft use; and (3) there was a positive association between county-level broadband access percentage and mental health video visits during compared to before the pandemic, but the strength of this association varied based on which broadband access measure was used. As health care systems continue to expand their use of video-based care, our findings highlight the importance of accurately defining and measuring broadband access from both research and clinical standpoints.

### Comparison With Prior Work

While telemedicine is known to increase access to care, studies have suggested that a patient’s ability to sufficiently access the internet may be a limiting factor [7,9,10,36,37]. Importantly, access to the internet is no longer the only defining feature of taking part in the digital age, but instead depends on broadband technology, speeds, and—at the individual level—economic cost, digital literacy, the number of simultaneous users, and other issues. For researchers, the measurement of broadband availability using FCC data is flexible regarding questions about varying speed levels and technology types (eg, digital subscriber line, cable modem, and fiber) and can be aggregated to a variety of census-based geographies. The largest hurdle to processing the FCC data is the sheer size of the files, although our study team has created a publicly available R (version 4.4.0; R Foundation for Statistical Computing) package to facilitate download and processing [38]. In comparison, Microsoft developed and released its broadband use data in a ready-to-use file, over which researchers have no control. Choosing which of these data sources is best suited to a particular research question will likely depend on the question of interest, the required control over additional broadband-specific features, the data management skills of the study team, and the desire to control for unmeasurable social determinants that influence internet access.

These findings also have clinical implications. After the onset of the COVID-19 pandemic, the VHA and other health care systems experienced a major shift to web-based mental health care. However, the most cited barriers to adoption were unreliable broadband, insufficient internet speeds, and technological barriers at the user level [39-42]. The VHA has attempted to address these barriers by providing internet-connected devices to patients meeting specific criteria [43], negotiating with telecommunication companies to provide free unlimited data while using VHA video telemedicine [44], and initiating test calls prior to a video visit to ensure the veteran knows how to use the VA Video Connect platform [45]. As health care systems and providers shift more of their care to video telemedicine, accurate measurement of broadband availability and access is crucial to improving health care system interventions adopted to close the digital divide, as well as the allocation of federal funding for the development of broadband infrastructure across the United States [46]. Targeting areas where infrastructure investment has been impacted by low population density (eg, fully rural counties) or geographic challenges (ie, mountains or valleys) may be a critical factor in improving equitable access to telemedicine [18]. Importantly, although increasing the availability of telemedicine opens additional avenues to care, limited access to adequate broadband may favor certain groups in unintended ways and worsen existing disparities among rural or already underserved populations [47].

### Limitations

This research has limitations. First, we did not assess causal relationships between video visit use and broadband percentages at the county level, nor did we account for patient comorbidities, familiarity with web-based care, or other factors related to telemedicine use. However, we highlight that we did adjust for age and other common sociodemographic factors that prior work has shown to be highly associated with telemedicine readiness and use [48]. Second, we were unable to distinguish when a video visit could not be completed, leading to a possible overcount of video visits. Third, this work focused only on mental health-based care. However, we contend that limitations in broadband access are likely generalizable to other types of care (eg, primary and subspecialty care), and thus we would expect similar findings if other types of care were assessed. Finally, data inadequacies, including our inability to account for cellular coverage areas may not accurately reflect those veterans using mobile versus terrestrial broadband connections.

### Conclusions

Despite limitations, this work is the first we are aware of to consider the use of FCC and Microsoft broadband data to triangulate the uneven impact of broadband access and the use of video mental health services. As health care systems expand video telemedicine, it is important to accurately measure where broadband infrastructure and related interventions can make the most impact and diminish access barriers to using this care modality. Future research should use the improved and more granular broadband data that are currently being collected by the FCC, investigate how differential broadband access may impact health care use, and whether increased access to care through video telemedicine has a beneficial impact on health care outcomes.

## Appendix

Multimedia Appendix 1 The protocol for calculating county-level penetration rates using Federal Communications Commission broadband data reported at the census block.

Multimedia Appendix 2 Percentage of veterans in the video use cohort and non–video use cohort within quintiles of 2019 penetration rate stratified by data set.

Multimedia Appendix 3 The estimation of enrolled veterans lacking adequate broadband for a video telemedicine visit by year and data source.

## Abbreviations

ADI: area deprivation index
FCC: Federal Communications Commission
IRR: incident rate ratio
Mbps: megabits per second
STROBE: Strengthening the Reporting of Observational Studies in Epidemiology
VA: Veterans Affairs
VHA: Veterans Health Administration
