# Supplementary material for: Comparing Federal Communications Commission and Microsoft Estimates of Broadband Access for Mental Health Video Telemedicine Among Veterans: Retrospective Cohort Study
Source: J Med Internet Res. 2024 Aug 8;26:e47100. doi: 10.2196/47100 (PMC11342002; doi:10.2196/47100)
Supplement: Multimedia Appendix 1 [file jmir_v26i1e47100_app1.pdf]

1. Identify at the census block, whether at least one broadband internet provider offered broadband speeds ≥25 Mbps download and ≥3 Mbps upload (i.e., “adequate” broadband), excluding satellite. This technology was excluded, because although widely available, it suffers from interference and delays, has relatively low subscription rates, and can be prohibitively expensive [11, 33, 34].
2. Determine overall population of each census block with Census Staff Block data.
3. Aggregate each census block to its corresponding county.
4. Sum the population of each census block meeting the adequate broadband requirement in Step 1 within each county. This creates the numerator of the county-level penetration rate.
5. Sum the population of all census blocks within each county. This creates the denominator of the county-level penetration rate.
6. Divide the county level numerator by the county level denominator and multiply by 100 to determine the county-level penetration rate as a percentage.
