# Supplementary material for: Comparing Federal Communications Commission and Microsoft Estimates of Broadband Access for Mental Health Video Telemedicine Among Veterans: Retrospective Cohort Study
Source: J Med Internet Res. 2024 Aug 8;26:e47100. doi: 10.2196/47100 (PMC11342002; doi:10.2196/47100)
Supplement: Multimedia Appendix 2 [file jmir_v26i1e47100_app2.pdf]

Table S1. Percentage of veterans in the video use cohort and non-video use cohort within quintiles of 2019 penetration rate stratified by dataset.

	Federal Communications Commission		Microsoft Broadband Usage	
	% Video Cohort	% No Video Cohort	% Video Cohort	% No Video Cohort
**2019 Penetration Rate Quintile**				
**5 (Better)**	29.5	70.5	31.0	69.0
**3**	29.6	70.4	25.6	74.4
**3**	25.3	74.7	23.9	76.1
**2**	23.4	76.7	22.0	78.0
**1 (Worse)**	21.7	78.3	18.7	81.3
**2020 Penetration Rate Quintile**				
**5 (Better)**	29.0	71.0	31.4	68.6
**3**	30.5	69.5	25.5	74.5
**3**	25.5	74.5	23.2	76.8
**2**	22.8	77.2	21.6	78.4
**1 (Worse)**	21.8	78.2	18.8	81.2
