# Supplementary material for: Comparing Federal Communications Commission and Microsoft Estimates of Broadband Access for Mental Health Video Telemedicine Among Veterans: Retrospective Cohort Study
Source: J Med Internet Res. 2024 Aug 8;26:e47100. doi: 10.2196/47100 (PMC11342002; doi:10.2196/47100)
Supplement: Multimedia Appendix 3 [file jmir_v26i1e47100_app3.pdf]

	Data Year	FCC Data<br>N (%)	Microsoft© Data<br>N (%)
**Total Number of Veterans**			
**19,226,221**			
	2019	1,595,558 (8.3%)	9,624,920 (50.1%)
	2020	1,411,708 (7.3%)	7,187,362 (37.4%)

This data was derived as follows:

1. Identify the 2020 county-level veteran population according to the VA National Center for Veterans Analysis and Statistics [30].
2. Subtract each county-level penetration rate from 100 to get the percent of the county without adequate broadband available.
3. Multiply the veteran population of each county from Step 1 times the penetration rate from Step 1 to estimate the number of veterans in each county without adequate broadband available. This assumes that veterans are evenly distributed throughout a county.
